# Human-to-dog transmission of SARS-CoV-2, Colombia

**DOI:** 10.1038/s41598-022-11847-9

**Published:** 2022-05-12

**Authors:** Ricardo Rivero, Evelin Garay, Yesica Botero, Héctor Serrano-Coll, Bertha Gastelbondo, Marina Muñoz, Nathalia Ballesteros, Sergio Castañeda, Luz Helena Patiño, Juan David Ramirez, Alfonso Calderon, Camilo Guzmán, Caty Martinez-Bravo, Ader Aleman, Germán Arrieta, Salim Mattar

**Affiliations:** 1 Instituto de Investigaciones Biológicas del Trópico (IIBT), Facultad de Medicina Veterinaria y Zootecnia, Universidad de Córdoba, Montería, Colombia; 2 Centro de Investigaciones en Microbiología y Biotecnología-UR (CIMBIUR), Facultad de Ciencias Naturales, Universidad del Rosario, Bogotá, Colombia; 3 Clínica Salud Social, Sincelejo, Sucre Colombia

**Keywords:** Virology, Phylogeny, Protein structure predictions

## Abstract

Severe Acute Respiratory Syndrome Coronavirus 2 (SARS-CoV-2), the causative agent of the current COVID-19 pandemic, has evolved to have a wide range of hosts, including non-human primates, wild and domestic animals. The ACE2 protein has a high level of conservation and is the common receptor invertebrate species for a viral infection to occur; this receptor could give rise to anthroponotic events. This article describes the first event of symptomatic transmission in Latin America from a human to a dog by the B.1.625 lineage of SARS-CoV-2. We found 21 shared mutations in the complete genomes of viral sequences from owners and dogs. Further phylogenetic and molecular analysis showed that 100% co-localization of the clade helps to understand human-animal transmission. Prediction of the Spike protein structure of the sequenced virus and docking analyzes showed that the E484K mutation in the receptor-binding domain (RBD) could contribute to the viral affinity of dACE2. Therefore, close contact between SARS-CoV-2-infected humans and pets should be avoided to prevent the emergence of novel mutations of public health importance from anthroponotic events.

## Introduction

Severe Acute Respiratory Syndrome coronavirus 2 (SARS-CoV-2), the etiological agent of the current COVID-19 pandemic is a novel virus belonging to the *Betacoronavirus* genus and it is genetically closer to bat coronaviruses than human SARS. For the reasons mentioned above, it is known as a viral zoonosis^1,2^. Research on transmission mechanisms of the viruses focuses on person-to-person contact. However, domestic animals' susceptibility to this virus is still uncertain^3^. To date, 11 complete SARS-CoV-2 genomes isolated from dogs have been reported in the Global Initiative on Sharing All Influenza Data (GISAID), from oropharyngeal samples^4^. The mechanism for viral infections depends on the binding between SARS-CoV-2 S Protein Receptor Binding Domain (RDB) and angiotensin-converting enzyme 2 (ACE2) receptor, which is crucial for infection since it allows the internalization of the virion into host cells^5^. Recently, several SARS-CoV-2 variants have emerged with an enhanced affinity towards human ACE2^6^. Considering that ACE2 receptors are present in several animal species, interspecies infections could arise from human-to-animal contact. However, the efficacy of the cellular union depends on the affinity of the viral RBD towards the host’s receptors^7,8^. Recently, several variants of SARS-CoV-2 have emerged with an augmented infectious capacity and neutralization-escape ability, these variants carry mutations in the *spike* protein such as N501Y, E484K, and K417T which have been described to have a relationship with higher transmissibility and resistance toward natural-induced and vaccine-elicited neutralizing antibodies^9–11^. In January 2021, a novel lineage identified as B.1.625 was reported in 5.8% of sequenced genomes in Colombia during the first trimester. Due to its rapid augment in prevalence and the identification of characteristic mutations in *Spike’s* N-terminal Domain (NTD), Receptor Binding Domain (RBD) and S1/S2 accounting for increased transmissibility, drug-resistance and antibody escape it was a rapidly growing lineage that circulated through America and Europe. The B.1.625 lineage is now circulating in 10 countries, including Colombia, and continues to pose a threat to public health and vaccine efficacy^12–14^. It is still unknown if the emergence of novel variants of concern and interest with augmented transmissibility account for enhanced infectivity of non-human hosts. Research of a more significant scale under the “One Health” approach is needed to assess the feasibility of direct human-to-animal transmission of SARS-CoV-2 in domestic environments to understand better the dynamics of the viral infections and their risk towards other species, including humans. This research performs viral genome analysis through next-generation sequencing of two SARS-CoV-2 clinical isolates from a dog and its owner. Given the relevance of this issue, this research aimed to perform a molecular, phylogenetic, and molecular docking approach to a case of SARS-CoV-2 human-to-dog transmission in Colombia.

## Results

### Clinical case description of the infected canine

**Figure 1:**
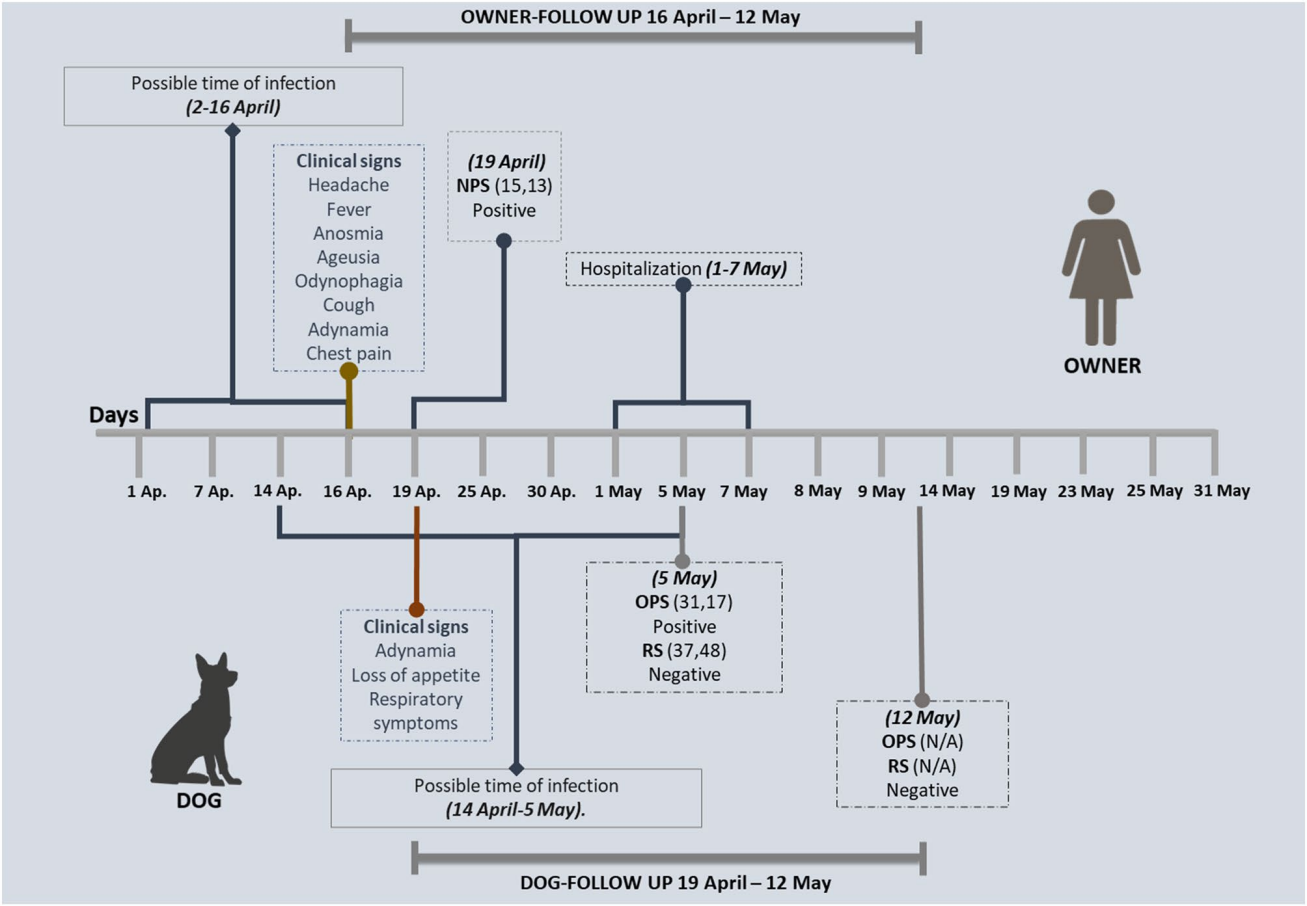
Timeline of symptom diagnosis, molecular testing, and follow-up.

A 54-year-old female presenting moderate clinical manifestations of COVID-19, such as fever, cough, headache, dyspnea, chest pain, and compromise of the lung parenchyma, tested positive for SARS-CoV-2 by RT-qPCR on April 19th, 2021, with a Ct = 15.13. Her two years old German shepherd dog presented sneezing, cough, hyaline rhinorrhea, diarrhea, vomiting, adynamia, and lack of appetite for three days after having frequent close contact with its unvaccinated owner, which was in knowledge of the research being done by Universidad de Cordoba and reached out to researchers for pet’s sample collection. RT-qPCR was conducted from oropharyngeal and rectal swab samples collected from the dog, resulting in a positive SARS-CoV-2 diagnostic by RT-qPCR (Ct = 31.36) on May 5th, 2021. A week later, a follow-up RT-qPCR test was conducted with a negative result in oropharyngeal and rectal swab samples. The animal did not show any other sign of illness after the negative result (Fig. 1). It is essential to mention that the dog had a strict indoor lifestyle and does not live with other pets. From the 290 domestic animals evaluated in this research study, 87.6% were felines and 12.4% were canines. RT-qPCR detected SARS-CoV-2 in a cat (EPI_ISL2339859.2) and a dog for total active infection rate of 0.69%.

### Molecular and phylogenetic characteristics of viral genomes

**Figure 2:**
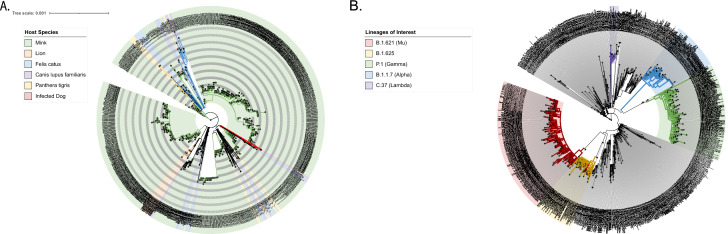
(**A**) Phylogenetic trees of sequences available in GISAID from minks, lions, tigers, cats, and dogs, U117 is highlighted in red. (**B**) Phylogenetic tree comprising Colombian sequences, VOC, and lineages of interest are annotated within the tree; U117 and human index case U118 are highlighted in red.

Two full-genome sequences labeled anonymously as U118 (EPI_ISL_8422792) and U117 (EPI_ISL_8422346) were generated from RNA samples of an infected dog and its owner. The resulting SARS-CoV-2 genomes were compared with the reference NC_045512.2-Wuhan-Hu-1, lineage assignment in Pango Lineages^15^ identified both sequences as part of B.1.625 lineage, which is located within the 20A clade and more specifically within the E484K sub-clade^16^ (Fig. 2). These genomes showed a 91% sequence similarity and shared significant mutations and deletions in M, N, ORF1a, ORF1b, ORF3a and S. B.1.625’s lineage defining T95I mutation was found in the viral spike protein among others such as E484K, D614G, N440K, as well as ∆H69-V70 and ∆Y144 deletions (Table 1), Phylogenetic analysis by maximum-likelihood co-located both sequences within a sub-clade with a support of 100 alongside Colombian B.1.625 sequences (Fig. 2), and Twenty-one out of 25 mutations found in U118 were also found within U117 viral genome, QC of obtained sequences confirmed a %Ns of 9.04 in dog sample, including a stretch of Ns located in S gene (21,147–21,386) (see Supplementary Data S1). Thus, it was confirming the transmission event between the human and its dog.

**Table 1:** Details of the SARS-Cov-2 genomes sequenced.

Host (species)	ID	Lineage	Mutations observed^a^
*Canis lupus familiaris*	U117	*B.1.625*	(n = 21)—**M** (I82T), **N** (M210I), **M** (234I); **ORF1a** (V86F), **ORF1a** (L681F), **ORF1a** (K1319E), **ORF1a** (T1822I), **ORF1a** (P2046S), **ORF1a** (K3353R), **ORF1a** (P3504S), **ORF1a** (∆S3675-F3677); **ORF1b** (K829R), **ORF1a** (P314L), **ORF1a** (T1076I); **ORF3a** (Q57H); **ORF8** (∆K2); **S** (F157L), **S** (N440K), **S** (E484K), **S** (D614G), **S** (D950N), **S** (V1228L)
*Homo sapiens sapiens*	U118	*B.1.625*	(n = 25)—**M** (I82T); **N** (M234I); **ORF1a** (V86F), **ORF1a** (L681F), **ORF1a** (K1319E), **ORF1a** (T1822I), **ORF1a** (P2046S), **ORF1a** (G2509D), **ORF1a** (K3353R), **ORF1a** (P3504S), **ORF1a** (∆S3675-F3677); **ORF1b** (K82R), **ORF1b** (P314L), **ORF1b **(T1076I); **ORF3a** (Q57H); **ORF7a** (I110V); **ORF8 (**∆K2); **S** (T95I), **S** (F157L), **S** (N440K), **S** (E484K), **S** (D614G), **S** (D950N), **S** (V1228L), **S** (∆H69-V70), **S** (∆Y144)

^a^In parentheses: amino acid substitutions/position aligned with reference NC_045512.2-Wuhan-Hu-1.

*N* nucleoprotein, *M* membrane glycoprotein M, *ORF* open reading frame, *S* spike protein.

Proteins with aminoacid substitutions are in [bold].

### Predicted structure of the isolated SARS-CoV-2 Spike protein and molecular docking of RBD and dACE2

**Figure 3:**
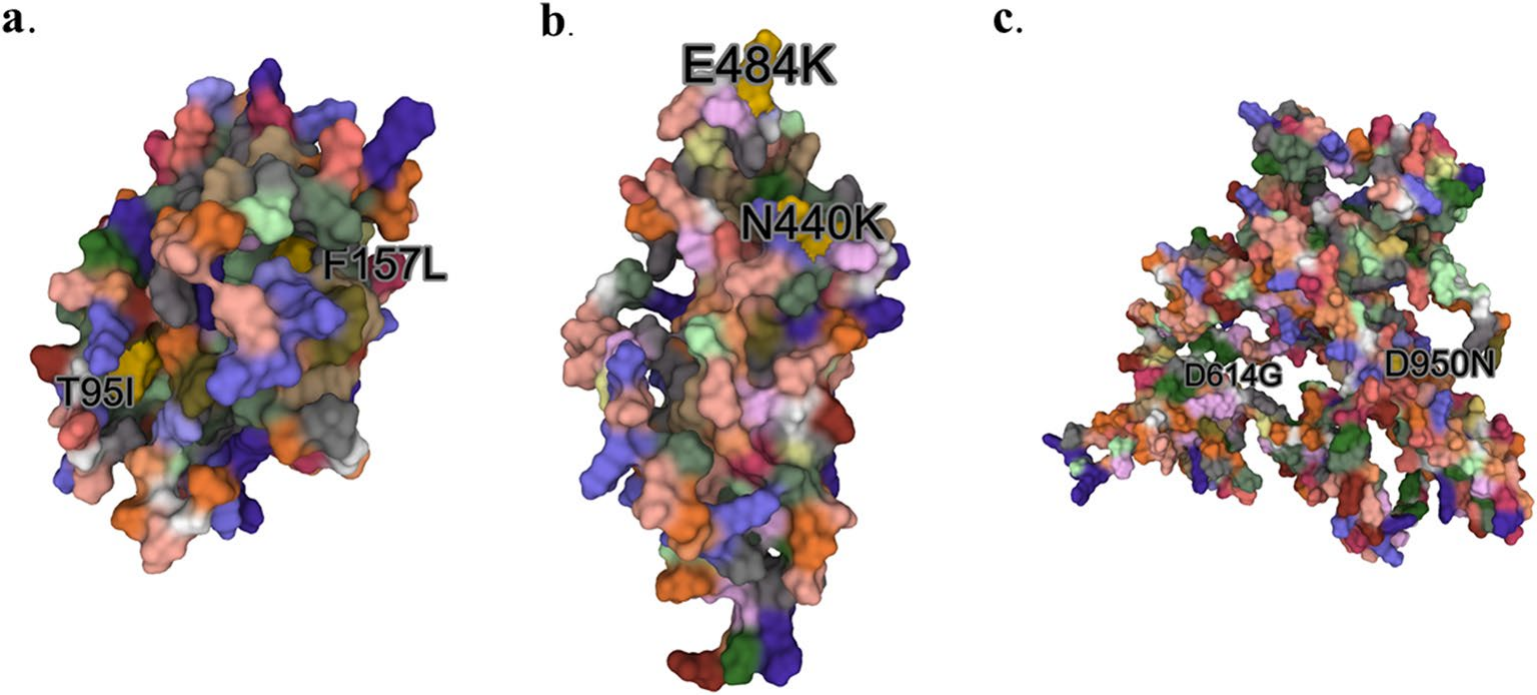
Predicted structure models of B.1.625 SARS-CoV-2 Spike Protein domains (**a**–**c**) N-terminal Domain, Receptor Binding Domain, and S1/S2 with mutations highlighted in yellow.

**Figure 4:**
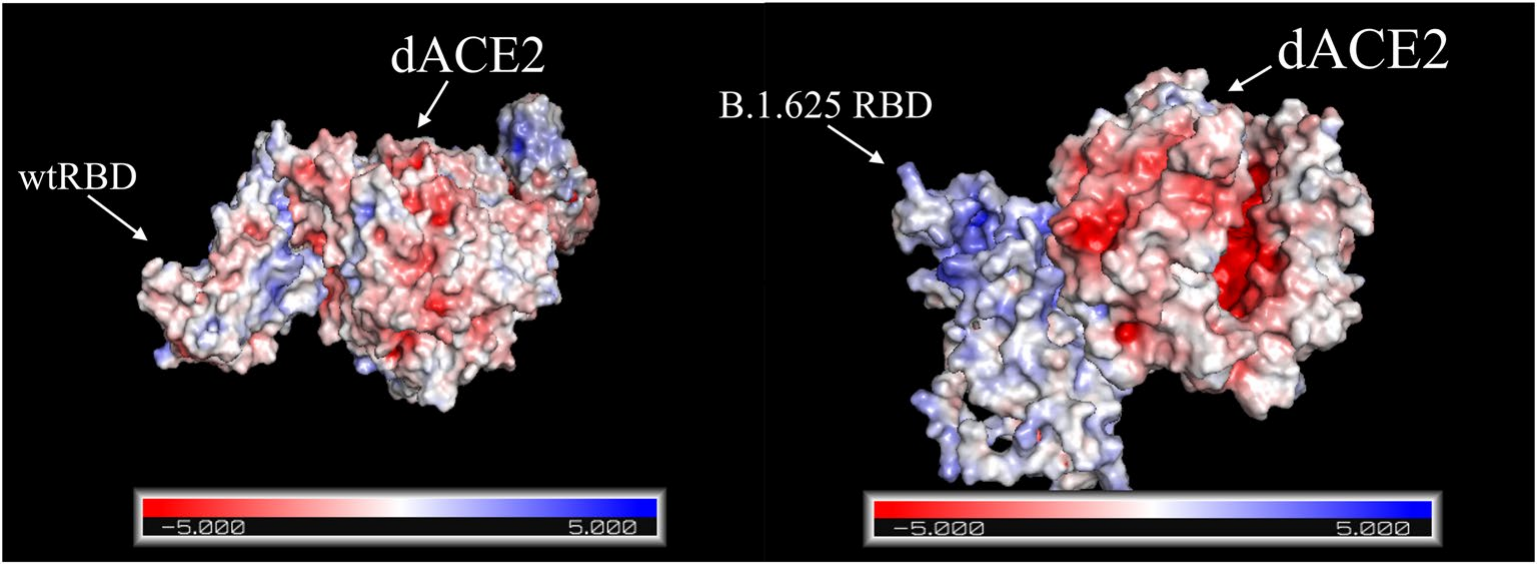
Electrostatic potential map of wtRBD-dACE2 and B.1.625 RBD-dACE2.

**Figure 5:**
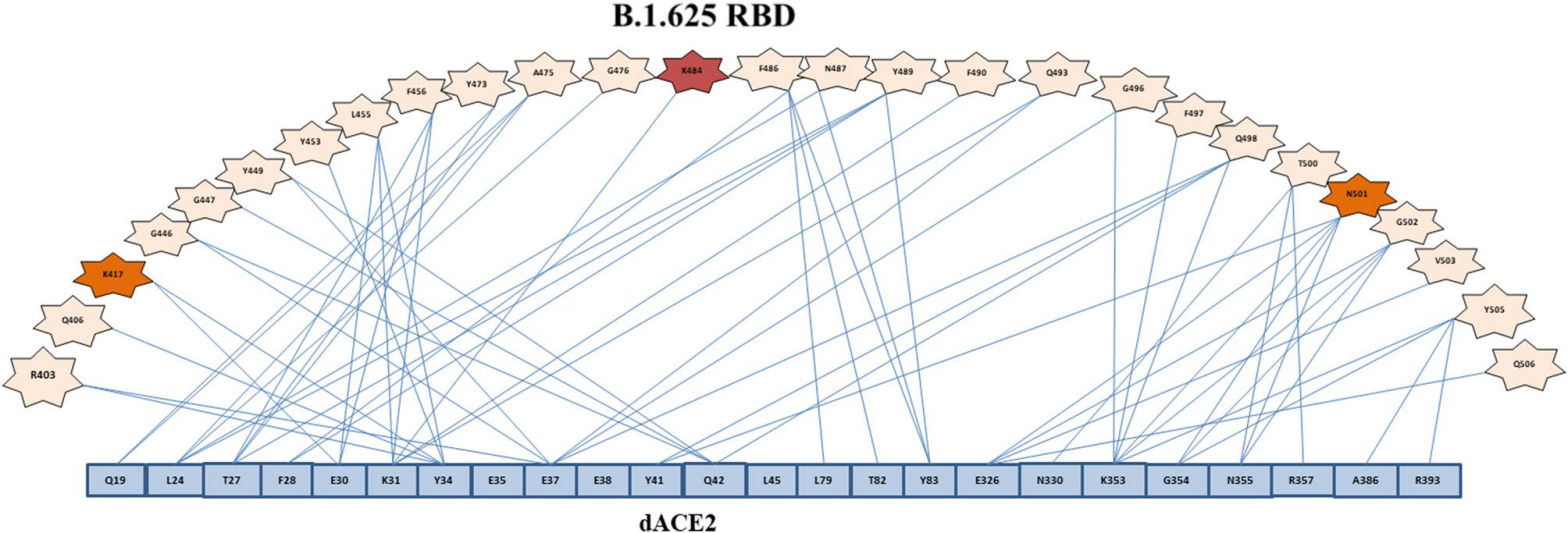
B.1.625 SARS-CoV-2-dACE2 interface residues. Mutation E484K is presented in red; residues 417 and 501 of importance in transmissibility enhancement are highlighted in orange.

Predicted 3D structures were generated from the sequence U118 for N-terminal domain, Receptor Binding Domain and S2 of the B.1625 S protein (Fig. 3). Docking analyses for B.1.625 RBD-dACE2 and wtRBD-dAC2 showed a standard free binding energy (ΔG°) of − 295.58 and − 264.11 kJ mol^−1^, respectively, which suggests a higher affinity of B.1.625’s RBD towards dACE2 compared to wild-type SARS-CoV-2 RBD. Also, electrostatic potential mapping evidenced that aminoacid substitutions found in B.1.625’s RBD predicted structure accounted for more positively charged residues when compared to wild-type protein, which could play a role in augmenting viral protein’s affinity towards the receptor (Fig. 4). Molecular docking analysis between B.1.625 RBD and dACE2, identified 27 interface residues within 5 Å distance including N501, K484 and K417 (Fig. 5).

## Discussion

Our results report the first case of human-to-dog transmission of SARS-CoV-2 in Latin America with successful isolation and sequencing of both viral full-genomes, and show concordance with previously reported studies on SARS-CoV-2 infection in dogs that had prolonged close contact with their owners^3,17^. A seroprevalence study carried out in Croatia found a 7.56% seropositivity in 172 randomly selected dogs living with healthcare, laboratory and veterinary personnel^17^. On the other hand, studies done in Spain and Italy could not obtain positive RT-qPCR from dog samples exhibiting pulmonary complications, but found a seroprevalence of 25% from dogs living in COVID-19(+) households, this indicates their susceptibility to SARS-CoV-2 infection and its relationship to close-contact with humans rather than dog-to-dog^7,18^.

An experimental infection carried by Bosco-Lauth et al.^8^ in cats and dogs that were inoculated with 3.0e5 and 1.45e5 PFU intranasally evidenced viral shedding occurring up to 5 days post-infection (DPI) in cats and moderate ulcerative, suppurative lympho- plasmocytic rhinitis in the nasal turbinates along with mild lymphoplasmacytic tracheitis but showed no signs of clinical disease, no weight-loss and body temperature < 39.5 °C, viral isolation was accomplished from trachea, nasal turbinates and esophagus of necropsied cats on day 5 post-infection. Contrastingly, no viral shedding nor clinical signs of disease was found in the experimental group of infected dogs with a seroconversion against RBD at 14 DPI^8^. In our study, although we did not perform viral isolation from samples, the upper respiratory tract clinical signs of disease observed in the infected dog could suggest active viral replication in lung tissue as well as in intestinal tract evidenced by the appearance of gastrointestinal signs such as vomiting, diarrhea, adynamia and lack of appetite.

The molecular and phylogenetic analyses of the obtained sequences support our hypothesis of a transmission event between the dog and its human owner, with both genomes being located within the same clade with a branch support of 100 and a total of 21 mutations shared between both sequences across isolated viral genomes. It is remarkable that these infections were due a Colombian SARS-CoV-2 lineage (B.1.625) which carries several mutations in the *spike* protein that confers augmented transmissibility and resistance to neutralization by naturally acquired and vaccine-elicited antibodies such as E484K mutation, which has been related to attenuation of anti-RBD neutralization^19^ and N440K has been observed to reduce viral susceptibility (up to 28-fold) to monoclonal antibody-based antiviral treatment^14^. This mutations reduces complementarity and electrostatic affinity between neutralizing antibodies and RBD, which could promote an enhanced viral immune evasion in both humans and animals, as well as an increase in reinfection cases and reduction the efficacy of anti-SARS-CoV-2 vaccines^12,20,21^.

Other spike mutations found in the obtained sequences such as N440K and D614G have been observed to reduce the efficacy of monoclonal antibody-based antiviral treatment up to 28-fold and augment the viral infectivity^14,22^. Furthermore, it is essential to emphasize that mutation D164G confers increased transmissibility to SARS-CoV-2 by stabilizing Spike protein, this prevents the S1 and S2 segments to be cleaved before RBD interacts with ACE2 receptor^23^. This could be associated with the greater affinity observed by Zhang et al.^24^ towards dog ACE2 (dACE2) of variants carrying D614G mutation when compared to Wu-1 SARS-CoV-2. Therefore, mutation D614G could play a key role for anthropozoonotic transmission of SARS-CoV-2^24^.

On the other hand, in vitro studies have reported that N501Y mutation found in several variants such as Alpha (B.1.1.7), Gamma (P.1) and Delta (B.1.617.2) enhances the affinity of RBD towards dACE2, accounting for a threefold increase in affinity (K_D_ = 37.1). Hence, wild-type RBD (K_D_ = 123 nM), augmented affinity and higher viral loads found in lineages carrying D614G mutation that was found in the present study, and it could cause an increase in human-to-animal transmission^22,24^.

The virological landscape in Colombia during the time of the study was characterized by the co-circulation of several lineages including Alpha, Gamma and Delta VOCs as well as Mu VOI which accounted for a percentage of 2.17, 12.8, 8.87 and 57.3 of all the reported sequenced between January and August of 2021, showing that the circulation of B.1.625 was very limited to the persistence and dominance of other lineages with enhanced transmissibility and immune escape^25–28^.

Zooanthroponosis has occurred in a mink farm in Denmark. this led to the culling of more than 17 million animals, and the emergence of a novel SARS-CoV-2 lineage with reduced susceptibility to antibody-neutralization (Mink cluster V)^6,29–31^. Due to the broad host range of SARS-CoV-2, self-isolation measures should be taken by humans to avoid the occurrence of zooanthroponosis events and public health risks which could lead to the emergence of novel lineages with new mutations of importance^32^.

Our study shows in silico that these mutations could account for an increase in the risk of zooanthroponosis events. However, in vitro experiments should be carried out for determining the affinity of VOC and VOI RBD towards animal hosts to assess the impact of the emergence of these novel and more transmissible lineages in non-human hosts.

In conclusion, the present study was the first reported case of a human-to-dog transmission event in Latin America supported by the sequencing and molecular characterization of both viral genomes. The identification of the B.1.625 lineage as the causative agent of this infection adds to the discussion on the importance of self-isolation measures between infected humans and pets. Considering the emergence of novel and more transmissible variants of SARS-CoV-2 could have an enhanced capability of infecting non-human hosts. Epidemiological and molecular surveillance of zooanthroponosis in pets co-habiting with SARS-CoV-2 patients is mandatory since it could represent an animal mutations risk for public health.

## Methods

### Sample collection and detection of SARS-CoV-2

Two hundred ninety samples were collected from domestic animals in 8 municipalities of the Cordoba department located in the north-western Colombian Caribbean, informed consent was obtained from human participants for sample collection in this study. Oropharyngeal and rectal swabs were collected for felines and canines as well as nasopharyngeal samples for humans as a part of the genomic and epidemiologic surveillance of pets living in COVID-19(+) patients’ households. Samples were conserved in a Viral Transport medium for SARS-CoV-2 detection by RT-qPCR. Briefly, RNA extraction was carried out using Thermofisher Genejet viral RNA/DNA extraction kit by following the manufacturer’s instructions for swab samples. Then, purified RNA was tested by RT-qPCR detection of SARS-CoV-2 E Gene following Charité Berlin protocol^33^. Those animal samples with a Ct value ≤ 32 was then subjected to next-generation sequencing of the virus’ complete genome. This study was approved by Universidad de Cordoba’s Veterinary Medicine faculty and its Ethics Committee number 005 (May 26th, 2021). All methods were carried out in accordance with national and international guidelines, including Law 84 of 1989 of the Congress of the Republic of Colombia, national guidelines of animal protection, resolution 8430 of Colombia’s Health Ministry and articles 87 and 88 of the 1989’s Universal declaration of Animal Rights. Additionally, animals and humans were sampled in compliance of CDC’s Guidelines for Safe Work Practices in Human and Animal Medical Diagnostic Laboratories and WHO Laboratory Biosafety Manual, ensuring human and animal wellbeing^34,35^. All methods were reported in accordance with the ARRIVE guidelines for the reporting of animal experiments^36^.

### Sequencing of RT-qPCR positive samples

Samples were subjected to whole-genome sequencing. Sequence libraries were prepared from RNA extracted from each nasopharyngeal/oropharyngeal swab per individual using the ARTIC Network protocol (https://artic.network/ncov-2019). Long-read Oxford Nanopore MinION sequencing was performed with the MinKNOW application (v1.5.5). Initially, raw Fast5 files were base called, and demultiplexed using Guppy; subsequently, reads were filtered by quality and length, eliminating possible chimeric and low-quality reads. Finally, genome assemblies were obtained following the MinION pipeline described in the ARTIC bioinformatics pipeline (https://artic.network/ncov-2019/ncov2019-bioinformatics-sop.html). Each assembly was typed based on the PANGOLIN nomenclature lineage allocator and SNPs identification was performed using the clade assignment, mutation calling and sequence quality checking tool, NextClade, which identifies differences between sequences and a reference sequence used by Nextstrain.

### Phylogenetic analysis of the sequences

Briefly, two datasets were generated from virus sequences reported in GISAID. For humans, 1855 viruses have been reported from Colombia in GISAID, incomplete sequences (< 29,000 bp) and sequences with low coverage (> 5% Ns) were excluded from the analysis resulting in a final dataset of 1002 sequences including the dog and owner. On the other hand, another dataset was created with sequences of SARS-CoV-2 detected in *Canis lupus familiaris* (dog), *Felis catus* (cat), *Panthera leo* (lion), *Panthera tigris* (tiger) and *Neovison vison* (mink) reported in GISAID globally by following the selection criteria as mentioned above to evaluate phylogenetic between globally reported sequences from cohabiting animals. The two datasets composed of a total of 1002 and 1057 sequences were subjected to multiple sequence alignment (MSA) against reference sequence NC_04551 in MAFFT v 7.48^37^ using “-addfragments”^37^ functionality within the webserver. Then, alignments were manually edited in Bioedit v.7.2.5 in order to add sample collection date to FASTA accession IDs as defined by REGEX (\d\d\d\d-\d\d-\d\d). Maximum-likelihood phylogenetic trees were reconstructed for each dataset in IQtree v.2.1.3^38^, ModelFinder^39^ was run for the datasets to determine the best-fit substitution model, this resulted in GTR + F + I being selected for tree reconstruction, branch support was calculated using UFBoot2^40^ within IQTree2. The consensus tree was visualized and tailored in FigTree v.1.4.4^41^ and rooted to NC_045512. Trees were exported in Newick format and then edited in iTOL^42^ for the addition of annotation schemes.

### Structural and molecular docking analysis of SARS-CoV-2 B.1.625 Spike protein

Predicted 3D models of *spike* protein domains, namely Amino-terminal Domain (NTD), Receptor Binding Domain (RBD), and S1/S2 were constructed in AlphaFold2^43^ from the sequence of SARS-CoV-2 B.1.625 lineage retrieved from the infected human, multiple sequence alignment for AlphaFold2 was done in MMseqs2^44^. Then, molecular docking analysis was carried out in HDOCK for comparing the affinity of wild-type SARS-CoV-2 RBD (wtRBD) (Protein Database accession ID 6M0J:B)^45^ and B.1.625 RBD towards both Human (hACE2) and dog (dACE2)^24^ angiotensin converting enzyme 2 (6M0J:A and 7E3J:A, respectively). Generated models for each interaction were ranked according to Standard binding free energy (ΔG°)^46^. Poisson-Boltzmann electrostatics visualizations were generated with PDB2PQR plugin within PyMOL^47^.

## Supplementary Information

Supplementary Information.
